# Use of a simple form to facilitate communication on long-term consequences of treatment in sarcoma survivors

**DOI:** 10.1186/s13569-019-0124-3

**Published:** 2020-01-16

**Authors:** Ivar Hompland, Lena Fauske, Geir Fagerjord Lorem, Øyvind S. Bruland

**Affiliations:** 1 Department of Oncology, Norwegian Radium Hospital, Oslo University Hospital, Nydalen, P.O. Box 4953, Oslo, 0424 Norway; 2 Department of Interdisciplinary Health Sciences, Institute of Health and Society, University of Oslo, Oslo, Norway; 3 Department of Health and Care Sciences, Faculty of Health Sciences, UiT The Arctic University of Norway, Tromsø, Norway; 4 Institute of Clinical Medicine, University of Oslo, Oslo, Norway

**Keywords:** Bone sarcoma, Soft tissue sarcoma, Late effects, Patient consultation, Follow-up, Fatigue

## Abstract

**Background:**

To report on our experience using a simple optional form to facilitate communication on late effects between the patients and the oncologists during outpatient follow-up and to detail on the spectrum of challenges reported by sarcoma survivors.

**Methods:**

The form was presented for the patients to complete before their consultation and covered topics related to late effects and unmet needs that the patient wished to discuss with the medical personnel. Logistic regression analysis examined how the distribution of the topics varied with age, gender, diagnosis and type of treatment received.

**Results:**

The form was manageable in a busy outpatient clinic. Of the 265 patients that received the form, 236 (89%) returned it. Patients in a palliative setting and those with other diagnosis than bone sarcoma (BS) and soft-tissue sarcoma (STS) were excluded for subsequent analyses. The final study-cohort comprised 160 patients, 54 (34%) with BS and 106 (66%) with STS. Among these, 140 (88%) had late-effect topics they wanted to discuss with their oncologist. Fatigue was raised by 39% of the patients, pain by 29% and impaired mobility by 23%. BS patients raised fatigue more often (*P *< 0.005) than those with STS. Patients who had undergone multimodal treatment with chemotherapy raised fatigue more frequently (*P* < 0.001) than those who had only undergone surgery, radiotherapy or both.

**Conclusions:**

A simple form on the long-term consequences of sarcoma treatment achieved a high response rate, was feasible to use in an outpatient clinic and facilitated communication on these issues. Fatigue was the most frequent topic raised and it was raised significantly more often in patients who had undergone chemotherapy.

## Background

A significant proportion of cancer survivors face a range of physical and psychosocial long-term consequences following treatment that negatively impact several aspects of their lives, leading to a general reduction in well-being [1, 2]. The need to address late effects of cancer treatment is widely acknowledged, as demonstrated by studies on conditions such as cancer-related fatigue, sexual dysfunction and infertility, pain, cognitive dysfunction, fear of recurrence, and disrupted body image [3–6]. However, the evidence shows that health care professionals are likely to underestimate or misjudge patient health preferences and support needs, especially among young and adolescent cancer survivors [7–9].

Treatment of patients with bone sarcoma (BS) and soft-tissue sarcoma (STS) often involves extensive surgery [10, 11] that can lead to physical and functional impairments [12, 13]. In addition to surgery, curative treatments often require a multimodal approach involving radiation and chemotherapy, significantly adding to the risk of therapy-related complications [10, 11]. Medical late effects include infertility, gonadal hormone deficiency, second malignant neoplasms, cardiomyopathy, pulmonary dysfunction, and renal insufficiency [14–17]. BS survivors, who are often adolescents and young adults [18], particularly struggle with late effects following treatment [13, 19, 20]. Health concerns among sarcoma survivors may be related both directly and indirectly to treatment. The patients’ perspective on the impact of late effects has not been thoroughly studied. Specifically, it is unknown which challenges patients consider most important for receiving support and guidance from health care providers at consultations during long-term outpatient follow-up.

A health intervention pilot project was initiated at Oslo University Hospital (OUH) after a previous study showed that bone cancer survivors reported considerable daily life challenges following cancer treatment [21, 22]. A simple form was introduced at our sarcoma outpatient clinic to facilitate communication between patients and oncologists regarding unmet needs. This form included well-known physical and psychosocial topics for patients to discuss with their clinicians to facilitate the patients’ need for information and guidance. The primary aim was to augment the quality of late-effect conversations by providing patients time before their consultations to ponder their most important complaints and by giving the oncologists insights and suggestions for topics to discuss with their patients. The present study reports on our experiences with this form. The secondary aim was to explore which topics BS and STS survivors considered the most important to discuss with their clinicians, and to determine whether there was a relationship between complaints related to diagnosis and the type of treatment received, as well as to explore any gender differences.

## Patients and methods

The Sarcoma Group at the Norwegian Radium Hospital, OUH, has a catchment area of approximately 2.8 million people and receive referrals also from other health regions in Norway. During systematic follow-up of patients treated at our institution, all demographic and disease-specific data are stored in a prospective clinical sarcoma database. The oncological sarcoma outpatient clinics take care of patients who have been treated with surgery and additional radiotherapy and/or chemotherapy. Systematic follow-up is usually undertaken for 10 years in expanding time-intervals with yearly consultations occurring after 5 years.

### Patients

**Fig. 1:**
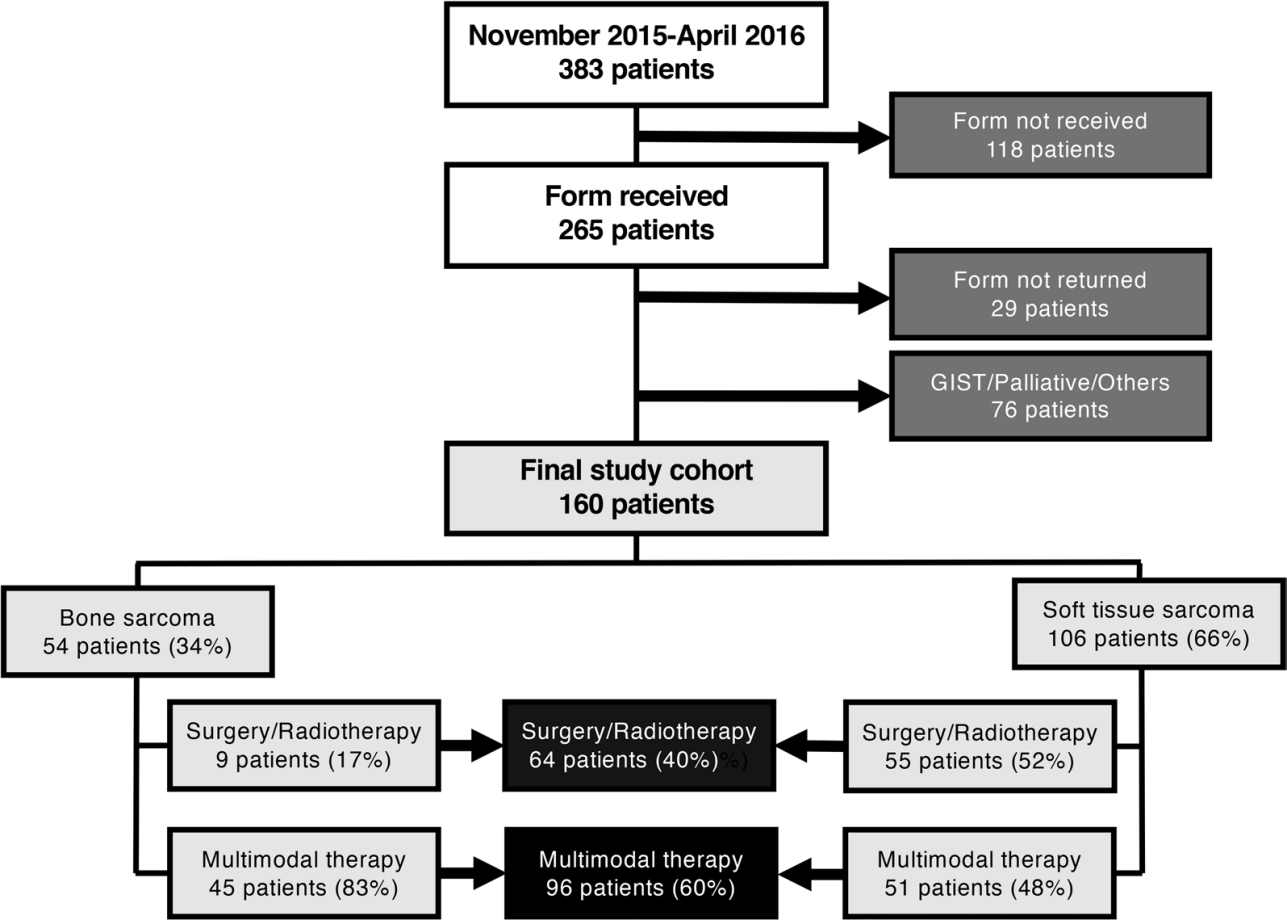
Consort diagram of the patients in the study

**Fig. 2:**
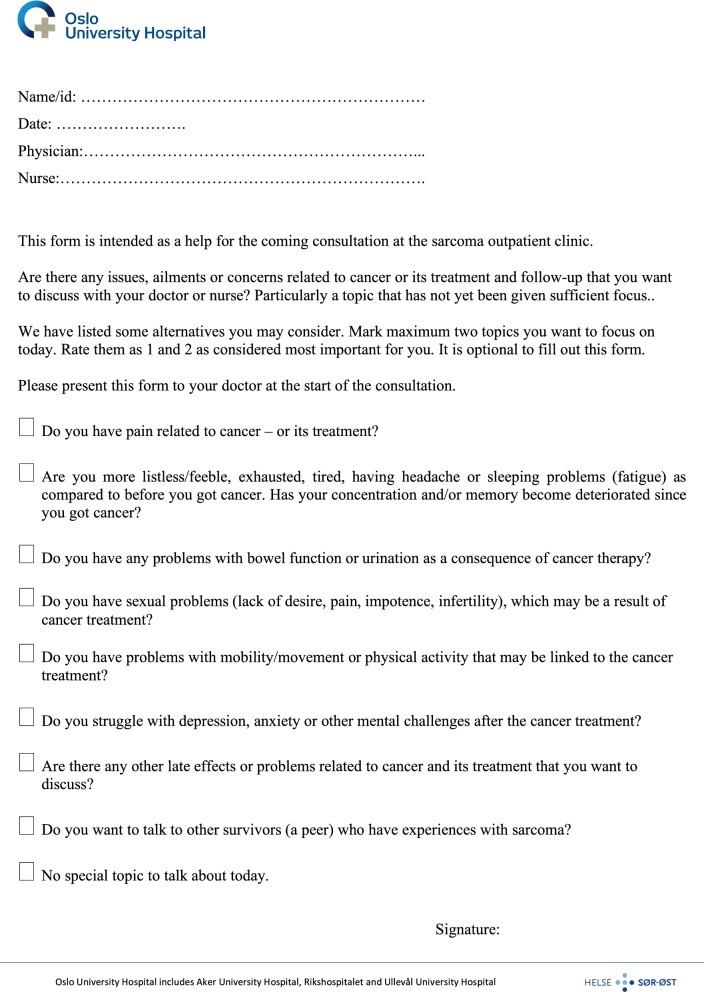
The form handed out to the patients. Translated from the Norwegian version into English

From the start of November 2015 to the end of April 2016, a total of 383 patients attended the two sarcoma outpatient clinics (Fig. 1). A form (Fig. 2) was presented to the patients as an optional service before their consultation and handed back to the clinicians at the start of the consultation. The form was not provided to any patients with evidence of metastases or local relapse or if the outpatient clinic that day was excessively busy (118 patients). The key information from the form was incorporated into the patient’s medical records. Demographic and disease-specific data were obtained from the prospective clinical sarcoma database to identify groups according to diagnosis, treatment (curative, long-term medical treatment and palliative) and age. This study was approved by the local data protection officer, and informed consent was obtained from all patients.

The present investigation focused on patients with STS and BS who had undergone treatment with a curative intent and who had not had any recurrence of the disease. The study excluded 76 patients with gastrointestinal stromal tumor (GIST), fibromatosis and other diagnoses, resulting in a final study-cohort of 160 patients (Fig. 1). These patients were stratified into two groups according to the type of treatment they had received. The first group included patients who had been treated with surgery only, surgery and radiotherapy, or radiotherapy only (SRT). The second group comprised patients who had received multimodal treatment including multi-drug chemotherapy (MMT) in addition to surgery and or radiotherapy.

### The form

A simple form (Fig. 2) to facilitate communication on late effects was constructed based on previous qualitative studies in BS survivors [21, 22]. The form covered seven topics related to late effects, one box that patients could mark if there was any other topic they wanted to raise with the clinician and one box that patients could mark if they did not have any specific topic to discuss (Fig. 2). The patients were encouraged to indicate a priority of up to two of these topics before the follow-up conversation with the clinician, and they were informed that such reporting was voluntary. The form was intended to prompt the doctor to secure time that day to respond to the topics prioritized by the patient. A process would then be initiated to provide general information, available printed materials, or referrals to other clinical specialists and resources within our hospital environment.

### Statistical analysis

Regarding the descriptive analyses of the demographic and clinic data, the Chi squared test or Fischer’s exact test was used where appropriate for categorical data. The Student’s *t*-test was used for continuous data (age). Associations between the different topics were analyzed using the phi coefficient (*Ø*). Logistic regression analysis was performed to examine how the distribution of the topics varied with gender, diagnosis (BS versus STS) and treatments (SRT versus MMT). Gender differences were adjusted for age, and the differences in diagnosis and treatments were adjusted for age and gender. The odds ratio (OR) and the adjusted OR are presented with 95% confidence intervals (95% CI). A two-tailed *P* value of less than 0.05 was considered statistically significant. All analyses were performed with the Statistical Package for the Social Sciences (SPSS) version 21 (SPSS, Inc., Chicago, IL).

## Results

### Patient demographics, diagnoses and treatments

The patient characteristics are summarized in Table 1. Of the 160 patients included in this study (Fig. 1), 74 were females, and 86 were males. The median age was 55 years (range 19–86). The study included 54 (34%) patients with BS and 106 (66%) with STS. The participants were divided into two groups: 64 (40%) who had undergone SRT and 96 (60%) who had undergone MMT. The median time from the end of a patient’s cancer treatment to the entering study entry was 6 years (range 1–31 years).

**Table 1:** Patients characteristics according to diagnosis and treatment groups

	Entire group, N = 160 (%)	BS, n = 54 (%)	STS, n = 106 (%)	*P*-value	SRT, n = 64 (%)	MMT, n = 96 (%)	*P*-value
Median age, years (range)	55 (19–86)	39 (19–80)	59 (19–86)	*< 0.001*	66 (28–86)	46 (19–78)	*<0.001*
Gender				0.09			0.94
Female	74 (46)	30 (56)	44 (41)		30 (47)	44 (46)	
Male	86 (54)	24 (44)	62 (59)		34 (53)	52 (54)	
Median time since diagnosis, years (range)	6 (0–31)	5 (1–22)	6 (1–31)	0.11	4 (1–19)	6 (1–31)	0.07
Time since diagnosis, years				0.80			*0.02*
<5	68 (43)	22 (41)	46 (43)		34 (53)	34 (35)	
≥5	92 (57)	32 (59)	60 (57)		30 (47)	62 (65)	
Treatment group				*< 0.001*			
SRT	64 (40)	9 (17)	55 (52)				
MMT	96 (60)	45 (83)	51 (48)				

Italic values indicate significance of *P*-value (*P* < 0.05)

*BS* bone sarcoma, *STS* soft tissue sarcoma, *SRT* surgery and or radiotherapy group, *MMT* multimodal treatment group

Comparing BS and STS, we observed that the median age was lower in the BS patients (*P *< 0.001). Also, the BS patients had undergone more MMT than the patients with STS (*P *< 0.001); the STS patients were more frequently treated with SRT and radiotherapy only. No significant differences were found related to gender (*P *= 0.08) or to the time since diagnosis (*P *= 0.80) (Table 1). The patients who had received MMT were younger (*P *< 0.001) and had a longer time since diagnosis (*P *= 0.02), but there were no differences in gender between the groups (*P *= 0.94) (Table 1).

### The form

The form was feasible to use in a busy outpatient clinic. It was not too time-consuming for the scheduled (20–30 min) consultations. The response rate was high, as 236 (89%) of the 265 patients who received the form returned it to the clinician (Fig. 1).

**Fig. 3:**
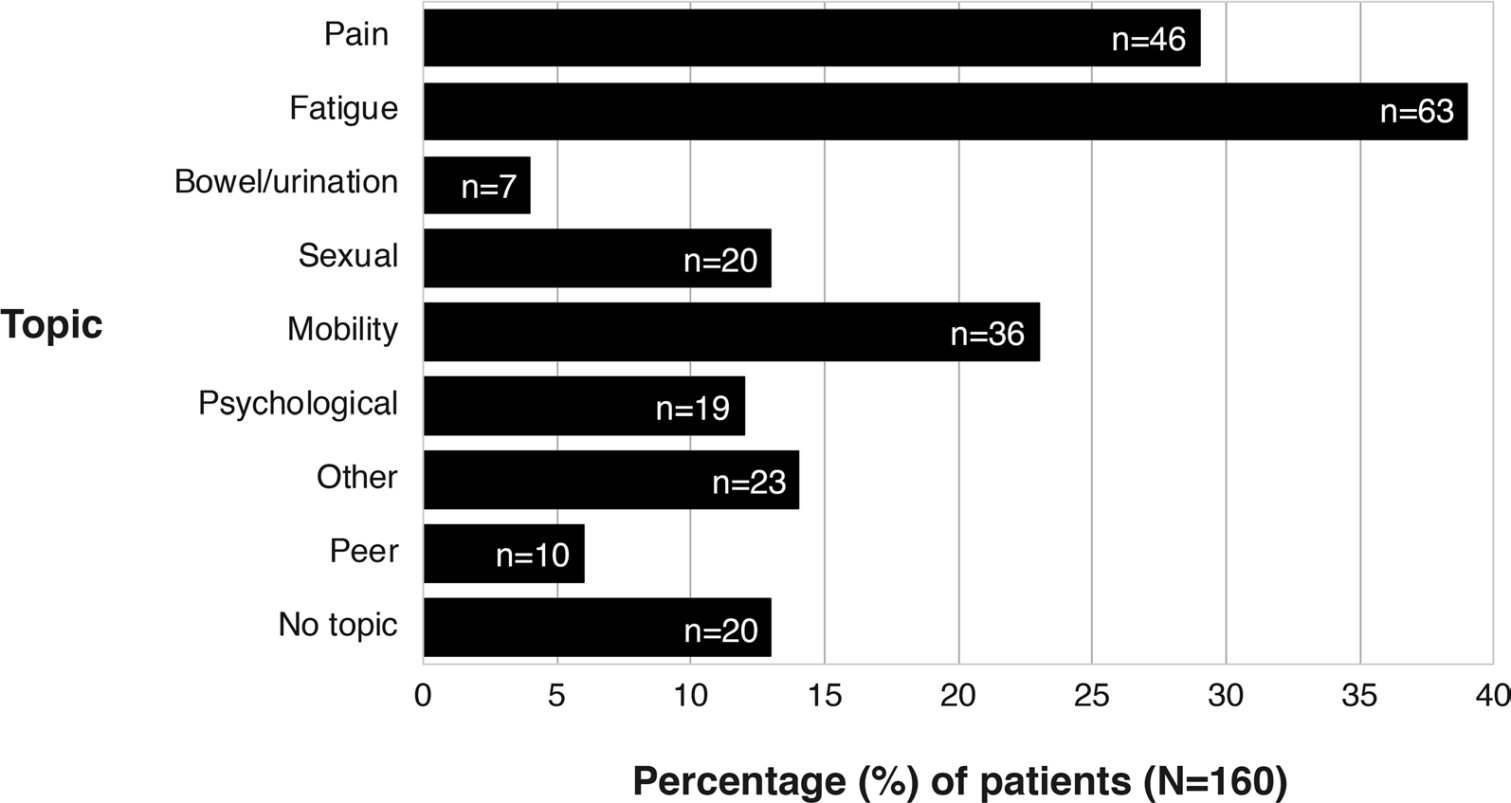
Distribution between the topics raised

In the patients who had undergone curative-intent treatment for BS and STS, 140 (88%) of the 160 patients had experienced late effects that they wanted to discuss with their clinicians. Only 20 patients (12%) reported no specific topic that they wished to discuss. Fatigue was raised by 39% of the patients, pain by 29%, and mobility challenges by 23%. The least used category was intestinal/urinary (4% of the patients) (Fig. 3). The patients who marked fatigue topic often wanted to discuss psychological issues (23% versus 4% who did not mark fatigue; *Ø *= 0.30; *P *< 0.001) and pain (40% versus 21% who did not mark fatigue; *Ø *= 0.22; *P *= 0.005). No other significant associations between the topics were found. There were no statistically significant gender differences in the use of the form (Table 2). However, a trend of males marking “no special topic to discuss” was found in both the non-adjusted (OR 2.7; 95% CI 0.9–8.5; *P *= 0.05) and adjusted analysis (OR 2.9; 95% CI 0.9–8.3; *P *= 0.05).

**Table 2:** Topic raised according to gender

Topic	Gender	N (%)	Crude OR (95% CI)	*P*-value	Adjusted OR^a^ (95% CI)	*P*-value
Pain	Female	24/74 (32)	1.0 (ref)		1.0 (ref)	
	Male	22/86 (26)	0.7 (0.4–1.4)	0.35	0.7 (0.4–1.5)	0.37
Fatigue	Female	32/74 (43)	1.0		1.0	
	Male	31/86 (36)	0.7 (0.4–1.4)	0.42	0.8 (0.4–1.5)	0.41
Bowel/urination	Female	4/74 (5)	1.0		1.0	
	Male	3/86 (4)	0.6 (0.1–2.9)	0.56	0.6 (0.1–3.0)	0.57
Sexual	Female	9/74 (12)	1.0		1.0	
	Male	11/86 (13)	1.1 (0.4–2.7)	0.90	1.1 (0.4–2.9)	0.83
Mobility	Female	15/74 (20)	1.0		1.0	
	Male	21/86 (24)	1.3 (0.6–2.7)	0.53	1.2 (0.6–2.8)	0.48
Psychological	Female	8/74 (11)	1.0		1.0	
	Male	11/86 (13)	1.2 (0.5–3.2)	0.70	1.3 (0.5–3.4)	0.63
Other	Female	15/74 (20)	1.0		1.0	
	Male	8/86 (9)	0.4 (0.2–1.0)	0.05	0.4 (0.2–1.0)	0.05
Peer	Female	4/74 (5)	1.0		1.0	
	Male	6/86 (7)	1.3 (0.4–4.8)	0.68	1.3 (0.3–4.8)	0.79
No topic	Female	5/74 (7)	1.0		1.0	0.05
	Male	14/86 (17)	2.7 (0.9–8.5)	0.05	2.9 (0.9–8.3)	

*OR* odds ratio, *CI* confidence interval, *ref* reference

^a^Adjusted for age

When the topics selected on the form were compared to diagnoses, we found that the patients with BS raised fatigue (OR 3.1; 95% CI 1.6–6.2; *P *< 0.001), sexual problems (OR 2.8; 95% CI 1.1–7.1; *P *= 0.04), psychological problems (OR 3.1; 95% CI 1.2–8.3; *P *= 0.02) and meeting a peer (OR 5.1; 95% CI 1.3–21; *P *= 0.02) significantly more often than the patients with STS (Table 3). Fatigue (OR 3.0; 95% CI 1.4–6.6; *P *< 0.005) and meeting a peer (OR 10; 95% CI 2.0–54; *P *= 0.005) remained statistically significant when adjusted for age and gender (Table 3). The patients in the MMT group raised the topic of fatigue (OR 4.3; 95% CI 2.1–8.8; *P *< 0.001) and mobility (OR 2.4; 95% CI 1.0–5.5; *P *= 0.04) significantly more often than the patients in the SRT group, but only fatigue (OR 4.3; 95% CI 1.9–9.5; *P *< 0.001) remained statistically significant in the adjusted analysis (Table 4).

**Table 3:** Topic raised according to type of sarcoma

Topic	Diagnosis	N (%)	Crude OR (95% CI)	*P*-value	Adjusted OR^a^ (95% CI)	*P*-value
Pain	STS	27/106 (26)	1.0 (ref)		1.0 (ref)	
	BS	19/54 (35)	1.6 (0.8–3.2)	0.20	1.5 (0.7–3.3)	0.36
Fatigue	STS	32/106 (30)	1.0		*1.0*	
	BS	31/54 (57)	*3.1 (1.6–6.2)*	*0.001*	*3.1 (1.4–6.7)*	*0.004*
Bowel/urination	STS	5/106 (5)	1.0		1.0	
	BS	2/54 (4)	0.8 (0.1–4.1)	0.77	0.6 (.01–4.1)	0.59
Sexual	STS	9/106 (8)	*1.0*		1.0	
	BS	11/54 (20)	*2.8 (1.1–7.1)*	*0.04*	2.6 (0.8–7.7)	0.10
Mobility	STS	20/106 (19)	1.0		1.0	
	BS	16/54 (30)	1.8 (0.8–3.9)	0.11	1.9 (0.8–4.5)	
Psychological	STS	8/106 (8)	*1.0*		1.0	
	BS	11/54 (20)	*3.1 (1.2–8.3)*	*0.02*	2.9 (0.9–8.5)	0.05
Other	STS	14/106 (13)	1.0		1.0	
	BS	9/54 (17)	1.3 (0.5–3.3)	0.56	1.4 (0.5–4.1)	0.50
Peer	STS	3/106 (3)	*1.0*		*1.0*	
	BS	7/54 (13)	*5.1 (1.3–21)*	*0.02*	*10 (2.0–54)*	*0.005*
No topic	STS	16/106 (15)	1.0		1.0	
	BS	4/54 (7)	0.4 (0.1–1.4)	0.17	0.5 (0.1–1.8)	0.27

N are presented as number of patients that raised the topic within the diagnosis group with % in the brackets

Italic values indicate significance of *P*-value (*P* < 0.05)

*STS* soft-tissue sarcoma, *BS* bone sarcoma, *OR* odds ratio, *CI* confidence interval, *ref* reference

^a^Adjusted for age and gender

**Table 4:** Topic raised according to type of treatment

Topic	Treatment group	N (%)	Crude OR (95% CI)	*P*-value	Adjusted OR^a^ (95% CI)	*P*-value
Pain	SRT	13/64 (20)	1.0 (ref)		1.0 (ref)	
	MMT	33/96 (34)	2.1 (0.9**–**4.3)	0.06	2.1 (0.9**–**4.8)	0.07
Fatigue	SRT	13/64 (20)	1.0		*1.0*	
	MMT	50/96 (52)	*4.3 (2.1–8.8)*	*< 0.001*	*4.3 (1.9–9.5)*	*< 0.001*
Bowel/urination	SRT	1/64 (2)	1.0		1.0	
	MMT	6/96 (6)	4.2 (0.5**–**36)	0.19	5.4 (0.5**–**54)	0.15
Sexual	SRT	6/64 (9)	1.0		1.0	
	MMT	14/96 (15)	1.7 (0.6**–**4.5)	0.33	1.3 (0.4**–**3.9)	0.68
Mobility	SRT	9/64 (14)	1.0		1.0	
	MMT	27/96 (28)	*2.4 (1.0–5.5)*	*0.04*	2.4 (0.9**–**5.8)	0.06
Psychological	SRT	6/64 (9)	1.0		1.0	
	MMT	13/96 (14)	1.5 (0.5**–**4.2)	0.42	1.0 (0.3**–**3.2)	0.96
Other	SRT	11/64 (17)	1.0		1.0	0.54
	MMT	12/96 (12)	0.7 (0.3**–**1.7)	0.41	05 (0.3**–**2.0)	
Peer	SRT	3/64 (5)	1.0		1.0	
	MMT	7/96 (7)	1.6 (0.4**–**6.4)	0.51	2.0 (0.4**–**9.8)	0.36
No topic	SRT	11/64 (17)	1.0		1.0	
	MMT	9/96 (8)	0.5 (0.2**–**1.3)	0.15	0.4 (0.1**–**1.3)	0.14

N are presented as number of patients that raised the topic within the treatment group with % in the brackets

Italic values indicate significance of *P*-value (*P* < 0.05)

*OR* odds ratio, *CI* confidence interval, *ref* reference, *SRT* surgery/radiotherapy group, *MMT* multimodal therapy group

^a^Adjusted for age and gender

As mentioned above, fatigue was moderately associated with psychological issues and pain. Hence, a multiple logistic regression analysis including these topics together with fatigue, age and sex was performed on the diagnosis and treatment groups to ascertain the significance of fatigue. In these analyses, fatigue remained statistically significant in both the diagnosis (BS: OR 2.7; 95% CI 1.2–6.0; *P *= 0.02) and treatment groups (MMT: OR 4.5; 95% CI 1.9–10.5; *P *= 0.001).

In the patients with STS, 51 (48%) underwent MMT, and 55 (53%) underwent SRT. The patients in the MMT group raised the topic of fatigue (HR 4.2; 95% CI 1.7–10; *P *= 0.002) significantly more frequently than the patients in the SRT group, even after adjustments for age and gender (HR 4.3; 95% CI 1.7–11; *P *= 0.003). No other differences were found between the treatment groups (data not shown). In the patients with BS, only nine (17%) patients received SRT, while 44 (83%) received MMT. Here, no statistically significant differences in the use of the form were found (data not shown).

## Discussion

In the present study, we observed that sarcoma survivors were eager to discuss their unmet needs and daily challenges with their clinicians. To our knowledge, this is first study to report a subjective ranking of late effects by sarcoma survivors in the context of discussing these issues with clinicians during long-term follow-up.

A high response was obtained from the form used in this study. Among the 160 patients included in this study, as many as 140 (88%) indicated a desire to discuss late effects with their clinician. Attention to this issue should be a vital part of the sarcoma patient follow-up. Our simple form was easy to use in a busy outpatient clinic and did not incur any additional costs. The patients were provided time to reflect on the topics included in the form, allowing them to focus on the conversation that followed. In our opinion, the form not only gave the doctor insight and suggestions for the follow-up conversations, it also increased the patient’s intake and comprehension of the conversation content. We hypothesize that this improved the quality of the consultation. In addition, the form could serve as a clinical survey for recognizing whether specific themes are repeated by patients that require special attention and action. As this was a pilot study with relatively few patients, this form should preferably be validated in a larger patient cohort. A validated, multidimensional, cancer-specific measure of unmet needs, such as the SCNS-SF34 [23], could have been used in this study. However, it is possible that this may not have encompassed needs which are unique to sarcoma patients. An obvious lack is the topic of mobility, since sarcoma survivors experience high rates of physical impairment  [12, 13]. To score subjective quality of life in patients who have undergone surgery for lower extremity malignant bone tumor, the Bt-Dux is often used [24]. This is, however, not necessarily applicable for patients who have undergone heavy chemotherapy. Also, both these instruments would probably have been too time-consuming to use in a busy outpatient clinic. Our results demonstrate that a simple communication form regarding late effects, such as the one used in this study, can draw attention to late effects for both the patient and the clinician and should be considered a routine part of follow-up consultations.

The most prevalent topic raised in the current study was fatigue (39%). Fatigue is known to have a multifactored etiology and pathogenesis [3], and it is one of the most commonly reported late effects following cancer treatment [25]. In sarcoma patients, one study found that fatigue was an important problem for more than one-fourth of the patients who had been treated for a malignant or benign bone or soft tissue tumor [26]. Fatigue is probably underreported by patients and underdiagnosed by clinicians [27]. The lack of a universally accepted definition for cancer-related fatigue likely contributes to this problem. The National Comprehensive Cancer Network has defined fatigue as “a distressing, persistent, subjective sense of physical, emotional, and/or cognitive tiredness or exhaustion related to cancer or cancer treatment, that is not proportional to recent activity and interferes with usual functioning” [28]. A similar description was used in our form. Although fatigue was not the main issue studied in this investigation and the questionnaire had not been validated for measuring fatigue, patient undergoing MMT (including chemotherapy), raised the topic of “fatigue-like complaints” significantly more often than patients who received only SRT. Patients wanting to discuss fatigue does not directly translate to a conclusion that the patients actually had fatigue. However, our results imply that fatigue may be more prevalent in sarcoma patients who have received chemotherapy as part of their treatment. This is further reinforced by our finding that BS patients raised the fatigue topic significantly more often than patients with STS. BS patients usually undergo more and longer chemotherapy than STS patients [10, 11], as was seen also in our cohort. When analyzing fatigue in the STS cohort and comparing the SRT and MMT groups, we found that the patient in the MMT group also raised the topic of fatigue significantly more often than the patients who only received SRT. These findings are at odds with the study by Servaes et al. [26] who found no differences in patients who received adjuvant therapy (including chemotherapy and radiotherapy). However, in that study, only 25 of 170 patients received adjuvant therapy and only 19 received chemotherapy, likely too small of a cohort from which to draw conclusions. Some research has shown that fatigue may be connected to a premorbid personality with neuroticism, poor communication and social functioning [29]. Higher emotional instability and self-reported stress in the premorbid period has been associated with a higher risk for chronic fatigue-like illness [30]. Across studies, the strongest and most consistent predictor of post-treatment fatigue is pre-treatment fatigue [3], but there is evidence that chemotherapy plays a role in fatigue development, as reported in a recent meta-analysis of breast cancer survivors [31]. Our findings support this notion and indicate that premorbid conditions should not be used an excuse to avoid justified guidance and support.

Fatigue and depression are strongly correlated in cancer [3], but it is difficult to determine whether fatigue is a symptom or a trigger of depression. Patients may attribute their depression symptoms to a less stigmatized state, e.g., as a somatic explanation that is translated into fatigue. Alternatively, fatigue may precipitate depressive moods by interfering with social, work-related and leisure activities. In the present study, the desire to discuss fatigue was moderately associated with raising the topic of psychological issues. However, when data were controlled for psychological issues, fatigue remained statistically significant. As for other previously reported studies [3], we cannot discern the causality between fatigue and depression. Nevertheless, a simple form such as the one used in our study may draw attention to both topics, revealing the nature of the complaints and guiding proper interventions.

Although fatigue awareness and the number of studies on the issue have increased in recent years, a focus on fatigue has not yet become routine during clinical follow-up [32]. According to the present findings, this practice should be adopted, especially in sarcoma patients who have received chemotherapy.

Pain (29%) and mobility (23%) were important topics to discuss for the patients. The patients in the present study attended orthopedic and abdominal sarcoma surgeons’ outpatient clinics in addition to our outpatient clinic. Hence, we cannot exclude that the patients focused on other topics at these oncological outpatient clinics and raised mobility topics more frequently with their surgeons. Interestingly, there were no statistically significant differences in the use of the form between genders. Whether the late effects of sarcoma treatment are universal to both genders cannot be answered by our study. There was, however, a trend toward males using “no topic to discuss” more frequently than females. In a larger patient cohort, this trend may have reached significance.

There are some limitations to the present study. As previously mentioned, the form used to score the late effects among patients and to facilitate communication between the clinician and the patient had not been validated. Since this was a pilot study integrated into a busy oncology practice, we chose to present the patients with a simple form. The majority of our patients responded to the form, and its use was manageable for the clinicians given the time available. To properly report on the issue of fatigue from a methodological perspective, a validated questionnaire [33] to score the symptoms and severity of fatigue would have been preferred. Due to the sample size and limited power, analyses of the interactions among the topics were not performed. The statistical power of the study was limited by the relatively small number of patients within the groups, implying a risk of type II statistical errors. Despite these limitations, we believe that our data accurately represent the clinical experiences of a busy oncological outpatient clinic.

## Conclusion

A simple form on the long-term consequences of sarcoma treatment achieved a high response rate, was feasible to use in an outpatient clinic and facilitated communication on these issues. Fatigue was the most prevalent topic raised by sarcoma patients, followed by pain and mobility. Fatigue was raised significantly more often among those who had undergone treatment with chemotherapy.

## Data Availability

The datasets generated during and/or analyzed during the current study are available from the corresponding author on reasonable request.

## Abbreviations

BS: bone sarcoma
STS: soft-tissue sarcoma
OUH: Oslo University Hospital
GIST: gastrointestinal stromal tumor
SRT: surgery and/or radiotherapy group
MMT: multimodal treatment group (including chemotherapy)
SPSS: Statistical Package for the Social Sciences
*Ø*: the phi coefficient
OR: odds ratio
CI: confidence interval
